# Measurement Properties of the BESTest Scale in People With Neurological Conditions: A Systematic Review With Meta-Analysis

**DOI:** 10.1093/ptj/pzae178

**Published:** 2024-12-12

**Authors:** Ilaria Arcolin, Marica Giardini, Federica Tagliabue, Valeria Belluscio, Fay Horak, Marco Godi

**Affiliations:** Department of Physical Medicine and Rehabiitation of Veruno Institute, Istituti Clinici Scientifici Maugeri IRCCS, Gattico-Veruno 28013, Italy; Department of Physical Medicine and Rehabiitation of Veruno Institute, Istituti Clinici Scientifici Maugeri IRCCS, Gattico-Veruno 28013, Italy; Department of Physical Medicine and Rehabiitation of Veruno Institute, Istituti Clinici Scientifici Maugeri IRCCS, Gattico-Veruno 28013, Italy; Department of Human Movement and Sport Science, University of Rome “Foro Italico”, Rome 00135, Italy; Department of Neurology, Oregon Health and Science University, Portland, OR 97239, United States; Department of Physical Medicine and Rehabiitation of Veruno Institute, Istituti Clinici Scientifici Maugeri IRCCS, Gattico-Veruno 28013, Italy

**Keywords:** Balance, BESTest, COSMIN, Measurements, Psychometrics, Rehabilitation

## Abstract

**Objective:**

People with neurological conditions (PwNC) frequently fall, mainly due to balance impairments. Among the scales assessing balance, the Balance Evaluation System Test (BESTest) is one of the most comprehensive in evaluating all components of postural control. This study aimed to systematically review and summarize the measurement properties of the BESTest in PwNC.

**Methods:**

Embase, MEDLINE, ScienceDirect, Scopus, and PEDro were searched up to December 2023. Studies assessing at least 1 BESTest measurement property in PwNC were included. Methodological quality of studies was assessed with the COSMIN Risk of Bias checklist. Overall rating and level of evidence for each property were given according to COSMIN criteria. Where possible, meta-analysis was performed.

**Results:**

Thirty-six studies (1749 PwNC) were included. The BESTest demonstrated a high quality of evidence supporting good reliability (intraclass correlation coefficients = 0.96–0.98 for total score, and 0.70–0.98 for subsections), internal consistency, and measurement error. High quality levels of responsiveness, and content and construct validity were also found. However, evidence for structural validity was insufficient to be sure the BESTest actually tests several, or 1, balance constructs. Criterion validity cannot be evaluated. While translated into different languages, cross-cultural validity has never been assessed in PwNC. Evidence to support use of the BESTest for specific neurological conditions is limited to Parkinson disease and stroke, due to the small sample sizes and number of studies in other populations.

**Conclusion:**

This systematic review provided high quality evidence supporting the reliability, content and construct validity, and responsiveness of the BESTest to intervention, being able to detect balance changes and to differentiate heterogeneous PwNC based on fall history, falling risk, and physical performance. However, low-quality evidence was found when considering each neurological condition alone. To comprehensively understand the BESTest measurement properties, future studies are needed with larger samples for each neurological condition, especially assessing cross-cultural and structural validity.

**Impact:**

Assessing balance is crucial for fall risk prevention. The BESTest has been demonstrated to be a reliable, responsive, and valid scale usable in clinical setting for assessing balance in PwNC.

**Lay Summary:**

Assessing balance is crucial for fall risk prevention. The BESTest has been demonstrated to be a reliable, responsive, and valid scale usable in clinical setting for assessing balance in PwNC.

## INTRODUCTION

Falls in people with neurological conditions (PwNC) are twice as frequent as in an age-matched community living population.^1^ Most falls result from a combination of intrinsic and extrinsic risk factors, with balance control deficits and antipsychotic medications considered the most prominent, predisposing factors.^2^

Balance is defined as the ability to maintain the body’s center of mass over its base of support (postural equilibrium) and to control body alignment with reference to itself and the environment (postural orientation).^3^ However, it is not a stand-alone skill, but requires a complex interaction of multiple sensorimotor processes^3^: (1) integration of information from somatosensory, visual, and vestibular systems; (2) automatic postural responses; (3) anticipatory postural adjustments; (4) voluntary limits of stability; (5) dynamic balance during gait; and (6) biomechanical structures and constraints to ensure balance. However, in the presence of a neurological disease, the level and the severity of the impairment of 1 or more of the balance processes can lead to partial or total loss of ability to safely perform daily life motor tasks (eg, walk, climb the stairs).^3^

Assessing and treating the physical parameters associated with falls is a complex task. A major strategy of many rehabilitation programmes for fall prevention has been the use of risk assessment tools to evaluate PwNC. Several studies have demonstrated that a battery of tests is necessary to fully assess balance as a fall risk factor.^4^ An issue commonly faced in balance assessment for clinical practice is that the majority of clinical instruments, including scales with good psychometrics, such as the Berg Balance Scale (BBS) or motor assessments like the Timed “Up & Go” test (TUG), are designed to test a single balance system or just few of the several balance domains (eg, Mini-Balance Evaluation Systems Test [Mini-BESTest]), and often do not specify which particular system of postural control is impaired. Identification of which specific balance system is impaired is important for the diagnosis of balance impairments and the development of individualized rehabilitation programs.^3^^,^^5^^,^^6^ As result, it is difficult to find a gold standard and a consensus on which assessment tool should be used in the clinical practice.^7^^,^^8^

Only few balance tests have been developed to identify underlying systems responsible for balance impairments. Among those, the BESTest^9^ scale is deemed to be the most comprehensive postural control scale able to assess individuals with different postural control capabilities.^10^

The BESTest includes 36 items evaluating performance across 6 balance systems: Biomechanical Constraints (I), Stability Limits and Verticality (II), Anticipatory Postural Adjustments (III), Postural Responses (IV), Sensory Orientation (V), and Stability in Gait (VI). Each item of the BESTest is scored on a 4-point ordinal scale ranging from 0 (severe impairment) to 3 (no impairment), for a total of 108 points. Since its introduction in 2009, the BESTest has been increasingly used for evaluating balance function in various populations – from elderly to people with neurological or musculoskeletal diseases.^11–13^ However, no systematic review synthetizing its measurement properties (ie, reliability, validity, and responsiveness) has been conducted.

Therefore, the aim of this study was to provide a comprehensive review of the psychometric features of the BESTest when administered to patients with balance dysfunctions caused by a variety of neurological diseases, to summarize the present evidence on this outcome measure, and to suggest gaps for further research.

## METHODS

### Data Sources and Searches

The COnsensus-based Standards for the selection of health Measurement INstruments (COSMIN) methodology and the Preferred Reporting Items for Systematic Reviews and Meta-Analyses were followed for conducting the current review.^14^^,^^15^ The present protocol was registered on PROSPERO (CRD42023407733).

Embase, MEDLINE, ScienceDirect, Scopus, and PEDro databases were searched since inception until December, 2023. Table S1 shows the search strategy. The reference lists of all included studies were screened for potential articles that might have been missed from the database search. Forward citation tracking of the included studies was conducted using Google Scholar.

### Study Selection

Studies were included if they met the following criteria:

- Design: randomized controlled trials, cohort studies, case-control studies, cross-sectional studies, and qualitative studies, published in peer-reviewed journals.
- Participants: ≥50% of the study’s population affected by a neurological disease.
- Intervention: studies reporting at least 1 of the psychometric properties of the BESTest scale included in the 3 domains (reliability, validity, and responsiveness) of the COSMIN taxonomy.^16^
- Outcome: BESTest.

Studies with no data on BESTest scale’s measurement properties, not peer-reviewed articles, as well as conference abstracts were excluded. Only English or Italian articles were considered.

### Data Extraction and Quality Assessment

Two independent reviewers (F.T., M.Gi.) carried out the systematic electronic searches. Both the initial screening of titles and abstracts for inclusion and the subsequent full text reading of the potentially relevant articles were autonomously performed by the same 2 authors. Controversial issues regarding inclusion/exclusion were resolved through discussion with a third author (I.A.).

Relevant data of the included studies (eg, authors, publication year, sample size, participants’ characteristics, study design, psychometric properties, relevant statistics) were extracted by M.Gi. and subsequently verified by I.A. for the final review. Data regarding measurement properties included content validity, structural validity (results of confirmatory factor analysis [CFA] or Rasch analysis), internal consistency (Cronbach alpha), reliability (intraclass correlation coefficients [ICC], Pearson or Spearman correlations), measurement error (standard error of measurement [SEM], minimum – or smallest—detectable change [MDC]), construct validity (Pearson or Spearman correlations for convergent validity; effect size and area under the receiving operating curve [AUC] for the discriminative or known-groups validity) and responsiveness to change (standardized response mean [SRM] or effect size in addition to Pearson or Spearman correlations and AUC).

For data synthesis, the 3 steps of the COSMIN guideline for systematic review of patient-reported outcome measures (PROMs) were followed:^17^

(1) The methodological quality of the studies was assessed using the COSMIN Risk of Bias checklist.^18^ The COSMIN checklist was composed by 10 boxes, 1 for each measurement property. Each item was rated on a 4-point rating scale as “excellent,” “good,” “fair,” “poor.” The overall quality score was obtained by taking the lowest rating on any of the methodological aspects according to the original COSMIN checklist.^16^

(2) COSMIN criteria for good measurement properties were applied to the results of the included studies.^15^^,^^18^ Each measurement property, in each individual study, was rated as sufficient (+), insufficient (−), or indeterminate (?), according to the predefined criteria. Ratings were then qualitatively summarized into an overall rating per measurement: when results of all the included studies were consistent, or at least 75% of the results were sufficient (or insufficient), the overall ratings were reported, with the summarized results rated as sufficient (or insufficient). Otherwise, if studies showed inconsistent results (ie, <75% of results supporting a particular conclusion), explanations for inconsistency were explored. For the analysis of construct validity and responsiveness of each single study, pre-defined hypotheses were formulated by the review team on top of the proposed COSMIN criteria (Table S2). When at least 75% of the studies’ results were in accordance with the hypotheses, the summary results of construct validity or responsiveness were rated as “sufficient.”

(3) The quality of the evidence of each measurement property was graded following a modified Grading of Recommendations Assessment, Development and Evaluation (GRADE) approach.^15^^,^^18^^,^^19^ The overall quality of evidence was rated as “high,” “moderate,” “low,” or “very low,” considering the risk of bias, inconsistency of study results, imprecision, and indirectness. Measurement properties previously rated as indeterminate did not receive any rating.

Each article was appraised separately by 2 researchers (F.T., M.Gi.). Discrepancies were discussed with a third researcher (I.A.) to reach mutual decisions.

### Data Synthesis and Analysis

A qualitative synthesis was performed to report study findings and methodological quality of each measurement property. The results were processed both in a heterogeneous way by grouping all the neurological populations enrolled, and individually by analyzing each neurological population separately. Meta-analyses were conducted on BESTest measurement properties when the results of a given measurement were clinically and statistically homogeneous across 5 or more studies. When correlation coefficients or ICC were analyzed, data were transformed to Fisher’s z scale and back-transformed for allowing interpretation of the results. Meta-analyses were performed using random-effects models with RStudio statistical software, version 4.2.2 (RStudio; Boston, MA, USA; http://www.rstudio.com/), using packages *meta*, *dmetar* and *tidyverse*. Studies were deemed to be statistically heterogeneous if *I*^2^ statistics exceeded 0.5. When a meta-analysis was inappropriate, the relevant measurement property was qualitatively summarized.

### Role of the Funding Source

The funders played no role in the design, conduct, or reporting of this study.

## RESULTS

**Figure 1:**
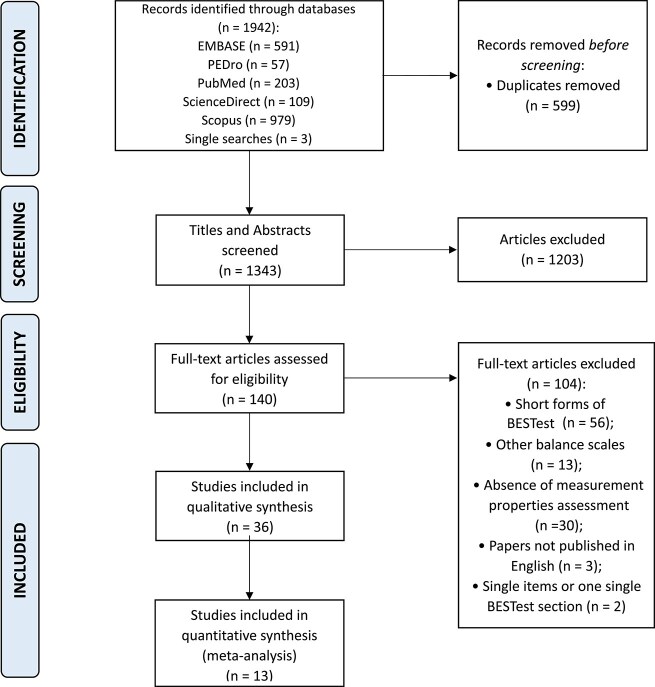
Preferred Reporting Items for Systematic Reviews and Meta-analyses (PRISMA) Flow Diagram of Study Selection. BESTest = Balance Evaluation System Test.

The electronic database search resulted in a total of 1942 citations, of which 599 were duplicates (Figure 1). From the 1343 studies screened using their title and abstract, 140 were selected for full text review. Overall, 36 articles were considered eligible, for a total of 1749 patients. Among those included studies, 12 studies involved patients with Parkinson Disease (PD),^20–31^ 10 studies recruited patients with stroke,^32–41^ 3 studies reported patients with multiple sclerosis,^42–44^ 3 reported patients with cerebral palsy,^45–47^ 1 studied patients with cervical spondylitic myelopathy (CSM),^48^ 1 studied patients with Alzheimer disease,^49^ 1 studied patients with spinocerebellar ataxia,^50^ 1 assessed patients with traumatic brain injury^51^ and, finally, 4 studies included patients with mixed neurological diseases.^9^^,^^52–54^

Characteristics of each study population are displayed in Table 1, while measurement properties’ results are reported in Table 2 and in Tables S3 and S4.

**Table 1:** Characteristics of the Studies Addressing the Measurement Properties of the BESTest^a^

**Population and Study**	**Country**	**Study Design**	**Sample Size, Total (F)**	**Age, y** **Mean (SD)**	**Testing Periods, d**
**Alzheimer disease**					
Tueth et al^49^ (2021)	US	Prospective cohort	25 (9)	76.4 (5.4)	365
**Cerebral palsy**					
Levin et al^45^ (2019)	US	Prospective cohort	20 (NA)	32.7 (9.3)	10
Morgan et al^46^ (2016)	Australia	Cross-sectional	17 (7)	37.0 (8.5)	NA
Opheim et al^47^ (2012)	Norway	Case–control	16 (11)	38.0 (6.7)	21
**Cervical spondylotic myelopathy**					
Chiu and Pang^48^ (2017)	China	Cross-sectional	72 (23)	63.9 (10.9)	3
**Mixed neurological diseases**					
Franchignoni et al^52^ (2010)	Italy	Cross-sectional	115 (62)	62.7 (16.0)	1
Hamre et al^53^ (2017)	Norway	Cross-sectional	22 (12)	66.5 (8.6)	4
Horak et al^9^ (2009)	US	Cross-sectional	14 (6)	67.1 (10.6)	540
Padgett et al^54^ (2012)	US	Cross-sectional	24 (11)	57.1 (13.0)	NA
**Multiple sclerosis**					
Jacobs and Kasser^42^ (2012)	US	Cross-sectional	13 (8)	50.0 (3.3)	NA
Mitchell et al^43^ (2018)	US	Cross-sectional	20 (16)	43.3 (10.2)	14
Potter et al^44^ (2018)	US	Observational	21 (16)	55.9 (9.6)	7
**Parkinson disease**					
Duncan et al^20^ (2012)	US	Prospective cohort	51 (30)	67.5 (8.8)	365
Duncan et al^21^ (2013)	US	Prospective cohort	80 (33)	68.2 (9.3)	365
Duncan et al^22^ (2015)	US	Prospective cohort	80 (33)	68.2 (9.3)	365
Duncan et al^23^ (2015)	US	Cross-sectional	78 (33)	68.1 (9.1)	NA
Leddy et al^24^ (2011)	US	Observational	80 (33)	68.2 (9.3)	16
Leddy et al^25^ (2011)	US	Cross-sectional	80 (33)	68.2 (9.3)	16
Maia et al^26^ (2013)	Brazil	Cross-sectional	35 (14)	66.5 (10.3)	7
Santos et al^27^ (2017)	Brazil	Prospective cohort	40 (8)	67.8 (7.2)	56
Santos et al^28^ (2023)	Brazil	Cross-sectional	55 (18)	67.0 (8.0)	1
Silva-Batista et al^29^ (2018)	US	Prospective cohort	39 (7)	64.2 (9.3)	84
Warlop et al^30^ (2016)	Belgium	Cross-sectional	20 (9)	65.3 (9.6)	NA
Wong-Yu et al^31^ (2015)	China	Prospective cohort	80 (34)	60.8 (9.0)	365
**Spinocerebellar ataxia**					
Kondo et al^50^ (2020)	Japan	Prospective cohort	20 (7)	63.7 (10.1)	30
**Stroke**					
***Subacute stroke***					
Chinsongkram et al^32^ (2014)	Thailand	Observational	82 (36)	57.2 (11.4)	7
Chinsongkram et al^33^ (2016)	Thailand	Prospective cohort	49 (20)	57.8 (11.8)	14
Hasegawa et al^35^ (2021)	Japan	Prospective cohort	30 (13)	76.4 (10.4)	79
Winairuk et al^41^ (2019)	Thailand	Prospective cohort	82 (30)	55.7 (12.3)	30
***Chronic stroke***					
Dadbakhsh et al^34^ (2023)	Iran	Cross-sectional	18 (3)	56.4 (9.0)	7
Miyata et al^36^ (2018)	Japan	Retrospective	140 (47)	70.0 (11.3)	7
Miyata et al^37^ (2018)	Japan	Prospective cohort	19 (8)	63.0 (13.3)	180
Miyata et al^38^ (2021)	Japan	Retrospective cross-sectional	87 (30)	70.2 (12.1)	NA
Rodrigues et al^39^ (2014)	Brazil	Cross-sectional	16 (3)	61.1 (7.5)	7
Sahin et al^40^ (2019)	Turkey	Cross-sectional	50 (20)	58.5 (17.0)	NA
**Traumatic brain injury**					
Hays et al^51^ (2019)	US	Cross-sectional	59 (21)	48.2 (12.3)	NA

^a^ BESTest = Balance Evaluation System Test; F = female; NA = not available; US = United States.

**Table 2:** Methodological Quality and Results of Studies on Measurement Properties of Responsiveness*^a^*

**Population and Study**	**Internal Responsiveness**	**External Responsiveness**	**COSMIN Score / Quality Score**
**Parkinson disease**			
Santos et al^27^ (2017)	ES = 0.49 for balance intervention group; ES = −0.11 for resistance training group. ES = 0.66 for between-group comparisons of the changes from baselines. Data for each section also available.	NA	Excellent (−) 1/2 hypotheses confirmed
Silva-Batista et al^29^ (2018)	ES = 1.04 for balance intervention group; ES = 0.24 for resistance training group. ES = 1.93 for between-group comparisons of the changes from baselines. r = 0.73 (*P* = .005) within BESTest and MoCA change scores. Data for each section also available.	NA	Good (+) 2/2 hypotheses confirmed
Wong-Yu et al^31^ (2015)	ES = 1.49 for balance intervention group; ES = 0.10 for upper limb training group. ES = 1.13 for between-group comparisons of the changes from baselines.	NA	Excellent (+) 2/2 hypotheses confirmed
**Stroke**			
***Subacute stroke***			
Chinsongkram et al^33^ (2016)	SRM (95% CI) = 1.2 (0.9–1.5).	**Anchor: BBS**: MCID = 10%, AUC (95% CI) = 0.92 (0.87–0.97), sensitivity = 0.81, specificity = 0.88, LR+/LR- = 6.5/0.2.	Fair (+) 2/2 hypotheses confirmed
Hasegawa et al^35^ (2021)	ES = 0.56, SRM = 1.28, RE = 0.99. Data for each section also available.	**Anchor: ambulatory independence at discharge**: AUC = 0.77, cutoff point = 16.7%, sensitivity = 0.73, specificity = 0.75.	Fair (+) 2/2 hypotheses confirmed
Winairuk et al^41^ (2019)	SRM = 1.23.	**Anchor: BBS*:*** MCID = 18.5, AUC (95% CI) = 0.89 (0.82–0.98), sensitivity (95% CI) = 0.79 (0.63–0.90), specificity (95% CI) = 0.94 (0.79–0.99), LR+/LR- = 12.63/0.22, posttest accuracy = 83%. **Anchor: GRC**: MCID = 8.5, AUC (95% CI) = 0.71 (0.53–0.88), sensitivity (95% CI) = 0.86 (0.74–0.94), specificity (95% CI) = 0.57 (0.29–0.82), LR+/LR− = 2.0/0.25, posttest accuracy = 77%.	Poor (+) 3/3 hypotheses confirmed

^a^ (+) = sufficient; (−) = insufficient. AUC = area under the receiver operating characteristic curve; BBS = Berg Balance Scale; BESTest = Balance Evaluation System Test; COSMIN = COnsensus-based Standards for the selection of health Measurement Instruments; ES = effect size; GRC = global rating of change; LR+ = positive likelihood ratio; LR− = negative likelihood ratio; MCID = minimal clinically important difference; MoCA = Montreal Cognitive Assessment; NA = not available; RE = relative efficiency; SRM = standardized response mean.

### Content Validity

Content validity and scale development were assessed in the study of Horak et al^9^ in a mixed neurological population (Table S3). The original BESTest publication reported a clear description of the construct to be measured, measurement aim, target population, and context of use. Being the methodological quality of this study classified as “good,” the content validity property was rated as “sufficient” with a high level of evidence (Table 3).

**Table 3:** Summary of Findings for Each Study Population and for All the Heterogeneous Populations Together About the Quality of Evidence Assessed With the 4 Items of the GRADE Approach and the Overall Rating of Each Measurement Error*^a^*

**Population**	**N**° **Studies**	**Content Validity**	**Structural Validity**	**Internal Consistency**	**Cross-Cultural Validity** ^***b***^	**Reliability**	**Measurement Error**	**Criterion Validity** ^***b***^	**Construct Validity**	**Responsiveness**
Alzheimer disease	1	NA	NA	NA	NA	NA	NA	NA	Low*^c^* (+)	NA
Cerebral palsy	3	NA	NA	NA	NA	Very low*^c^**^,^**^d^* (+)	Very low*^c^**^,^**^d^* (+)	NA	Low*^c^* (−)	NA
Cervical spondylotic myelopathy	1	NA	NA	Moderate*^e^* (+)	NA	Very low*^c^**^,^**^e^* (+)	Very low*^c^**^,^**^e^* (+)	NA	Very low*^d^**^,^**^f^* (+)	NA
Mixed neurological diseases	4	High (+)	Very low*^g^* (−)	Low*^c^* (+)	NA	Moderate*^f^* (+)	Low*^c^* (+)	NA	Moderate*^f^* (+)	NA
Multiple sclerosis	3	NA	NA	Low*^c^* (+)	NA	Very low*^c^**^,^**^e^* (+)	(?)	NA	Low (−)	NA
Parkinson disease	12	NA	(?)	NA	NA	Very low*^c^**^,^**^e^* (+)	Very low*^f^**^,^**^g^* (+)	NA	High (+)	High (+)
Spinocerebellar ataxia	1	NA	NA	NA	NA	Very low*^c^**^,^**^d^* (+)	Very low*^c^**^,^**^d^* (+)	NA	NA	NA
Stroke - subacute	4	NA	NA	NA	NA	Very low*^c^**^,^**^d^* (+)	Very low*^c^**^,^**^g^* (+)	NA	High (+)	Low*^d^* (+)
Stroke - chronic	6	NA	Very low*^g^* (−)	Very low*^f^**^,^**^g^* (+)	NA	Very low*^c^**^,^**^e^* (+)	Very low*^c^**^,^**^e^* (−)	NA	High (+)	NA
Traumatic brain injury	1	NA	NA	NA	NA	NA	NA	NA	Moderate*^f^* (+)	NA
**Heterogeneous populations**	**36**	**High (+)**	**Low** ^***d***^ **(−)**	**High (+)**	**NA**	**High (+)**	**High (+)**	**NA**	**High (+)**	**High (+)**

^a^ (+) = sufficient; (−) = insufficient; (?) = indeterminate; GRADE = Grading of Recommendations Assessment, Development and Evaluation; NA = not available.

^b^ Data are not available due to the absence of studies investigating these measurement properties.

^c^ Downgraded for imprecision, sample size <50.

^d^ Downgraded for very serious risk of bias.

^e^ Downgraded for serious risk of bias.

^f^ Downgraded for imprecision, sample size 50 < 100.

^g^ Downgraded for extremely serious risk of bias.

### Structural Validity

Three studies^26^^,^^36^^,^^52^ assessed the structural validity of the BESTest in people with stroke and mixed neurological diseases (Table S3). Two studies performed the CFA,^36^^,^^52^ while 1 study used the Rasch model.^26^ Both CFA studies demonstrated poor methodological quality, mainly due to inadequacy of the sample size used for assessing the CFA. In fact, the number of participants included in these studies was below the cut-off of 5 times the number of items of the BESTest (=180). In both the studies of Franchignoni et al^52^ and Miyata et al,^36^ the fit indices for the 36-item unidimensional model of the BESTest were poor (Table S3). Conversely, the fit indices of the original 6-factor model for the 6 domains of balance control proposed by the developers^9^ (biomechanical constraints, stability limits and verticality, anticipatory postural adjustments, postural responses, sensory orientation, and stability in gait) were better compared to the unidimensional model. The 25 item, 4-factor model of the BESTest proposed by Miyata et al^36^ and composed of dynamic postural control during gait, standing postural control, compensatory stepping reactions, and stability limits, obtained acceptable fit indices. Moreover, each domain of the 25-item model showed a high degree of unidimensionality. Finally, the study^26^ evaluating the structural validity using the Rasch model showed a poor methodological quality due to its small sample size (<100 participants) and the inaccuracy in the methods used for conducting the Rasch analysis. Based on review of these 3 studies, evidence that the BESTest represents a unidimensional construct of “balance control” was rated as “insufficient” (Table 3).

### Internal Consistency

Four studies^40^^,^^44^^,^^48^^,^^54^ evaluated the internal consistency of the BESTest scale in 4 different patient groups (Table S4). One study^40^ was classified as poor methodological quality because it calculated the Cronbach alpha only for the total score, without assessing or providing evidence that the questionnaire was unidimensional. On the contrary, the other 3 studies^44^^,^^48^^,^^54^ were rated as excellent, as internal consistency was evaluated for each subscale of the BESTest. In all these studies, the Cronbach alpha of the subscales were > 0.70, except for the Section II in the study of Padgett et al.^54^ In patients with CSM, Cronbach alpha of BESTest subscales ranged from 0.85 to 0.98^48^ and in patients with multiple sclerosis from 0.66 to 0.93.^44^ Based on these findings, the evidence on internal consistency was rated as “sufficient” since >75% of the values were good for the original subscales, assuming that these subscales were unidimensional, even if this has not yet been proven with factor analysis. Quality of evidence for the heterogeneous populations was graded as “excellent,” with most of the studies of “excellent” quality (Table 3). However, when considering each single study population, evidence was “moderate” in CSM, and “low” or “very low” in the other study groups, mainly due to the limited sample enrolled in each study population.

### Reliability

Thirteen studies assessed the reliability of the BESTest in 8 different populations^9^^,^^25^^,^^26^^,^^32^^,^^34^^,^^39^^,^^43–45^^,^^48^^,^^50^^,^^53^^,^^54^ (Table S4). Eight studies investigated the test–retest reliability, 7 studies examined the interrater reliability, and 2 studies included the intrarater reliability of the BESTest. Methodological quality of 2 studies^9^^,^^53^ was rated as “excellent,” 4^34^^,^^44^^,^^48^^,^^54^ as “good,” and 7^25^^,^^26^^,^^32^^,^^39^^,^^43^^,^^45^^,^^50^ as “fair.” The lack of information regarding the stability of patients or the test conditions, as well as the absence of descriptions of the ICC formula used, buted to the low methodological quality scores. All the studies assessing reliability reported ICC values ≥0.70 and were rated as sufficient. While all 13 studies reported the ICC value for the BESTest total score, only 9 assessed the ICC of the subscores. Based on these findings, the evidence on reliability was rated as “sufficient,” with a global quality of evidence graded as “moderate,” because more than half of the studies were found of “fair” quality. The pooled values of reliability are reported in Figures S1, S2, and S3. Test–retest reliability was found “excellent” with an ICC for the total score of 0.96 (95% CI, 0.91–0.98; *P* < .01; τ2 = 0.23; Figure S1A), while ICC for the 6 subsections of the BESTest ranged from 0.70 (95% CI, 0.61–0.77; *P* = .24; τ2 = 0.01; Figure S2) for the Section II to 0.91 (95% CI, 0.81–0.96; *P* < .01; τ2 = 0.19; Figure S2) for the Section VI. Interrater reliability was “excellent” for total BESTest score, with an ICC of 0.98 (95% CI, 0.95–0.99; *P* < .01; τ2 = 0.16; Figure S1B), while “good” for all subsections, with ICC values above 0.80 (Figure S3). Although the overall quality of evidence was “high” for the patient groups considered together, reliability’s quality of evidence for each population alone was rated as “very low,” due to the limited sample of patients enrolled in each study population (Table 3).

### Measurement Error

The measurement error was assessed in 9 studies assessing 6 different patient groups included in this review^29^^,^^34^^,^^41^^,^^43–45^^,^  ^48^^,^^50^^,^^52^ (Table S4). Measurement error was commonly calculated from the test–retest reliability and expressed as number of points on the BESTest. Three studies^34^^,^^44^^,^^48^ showed a “good” methodological quality, 3 studies^43^^,^^45^^,^^50^ reported a “fair” quality, and 2 studies^29^^,^^41^ reported a “poor” quality. The main reason for fair and poor methodological quality was the non-stability or the absence of clear information regarding the stability of patients. The SEM was assessed in only 5 studies, with a range between 1.15 and 8.33. The MDC_95_ was calculated in all the 9 studies, with a value ranging between 3.19 and 22.82, with the value of 4.96 being identified by the study of excellent quality. Moreover, 5 studies also reported the MDC_95_ for each subscale of the BESTest. As reported by COSMIN guideline,^18^ the criteria for assessing the quality of rating of measurement error property is based on the comparison between MDC and the minimal clinically important difference (MCID). The latter has been calculated with the anchor-based method in 3 studies of fair and poor-quality enrolling patients with stroke^33^^,^^35^^,^^41^: the triangulation of their results led to a MCID of 11.9 points. Based on these findings, the property measurement error was rated as sufficient, since only 2 studies reported a MDC_95_ value over the threshold of the MCID, while the quality of evidence was graded as “high” having 1 study rated as excellent (Table 3). As for reliability, the quality of evidence of measurement error was “low” or “very low” when considering each study population alone.

### Criterion Validity

Criterion validity was not evaluated due to the lack of a gold standard criterion.^18^

### Cross-Cultural Validity

The BESTest has been translated in to at least 9 languages,^55^ but few studies presenting its translational and cross-cultural adaptation have been published.^26^^,^^34^^,^^53^^,^^56–59^ Four of these studies^56–59^ were excluded due to the inclusion criteria (not being in English and the study populations). Although the remaining cross-cultural studies included an independent, back-translation by the original author of the BESTest,^26^^,^^34^^,^^53^ they did not assess whether the performance of the items on a translated BESTest were an adequate reflection of the performance of the original, English BESTest. Therefore, no evidence about the cross-cultural validity of the BESTest in PwNC can be given.

### Construct Validity

Construct validity was assessed in 26 studies^9^^,^^20–25^^,^^28^^,^^30^^,^^32^^,^  ^36–44^^,^^46–49^^,^^51^^,^^53^^,^^54^ (Table S3). Among those, 20 studies investigated the convergent validity, while 14 studies evaluated the discriminative or known-groups validity. Most of the studies evaluating convergent validity assessed the correlations with other commonly used balance scales, such as the BBS, Brief-BESTest or Mini-BESTest. When considering the known-groups validity, 8 studies investigated the ability of the BESTest in discriminating fallers from nonfallers, 4 studies in identifying those with or without a falling risk, and 5 studies in differentiating people with different physical performances. Since no studies described any *a priori* hypotheses for assessing construct validity, the review team used their preformulated hypotheses. Methodological quality of 13 studies^9^^,^^22^^,^^25^^,^^28^^,^^30^^,^^32^^,^^37^^,^^39^^,^^41^^,^^47^^,^^49^^,^^51^^,^^53^ was rated as “excellent,” 6^21^^,^^23^^,^^24^^,^^36^^,^^38^^,^^40^ as “good,” 5^20^^,^^42^^,^^43^^,^^48^^,^^54^ as “fair” and 2^44^^,^^46^ as “poor.” The main reasons for low methodological quality were that the distributions of scores of the comparator instruments were not shown, that it was unclear whether the comparator instruments had sufficient measurement properties in the study population, or there was no clear description of the important characteristics of the subgroups. Of the 26 studies, 21 were rated as “sufficient,” while 5 as “insufficient.” Therefore, since 85% of the results of the 26 studies were in accordance with the preformulated hypotheses, the overall rating of the construct validity was “sufficient,” with a quality of evidence graded as “high” (Table 3). When analysing the study populations separately, it emerged that construct validity was “sufficient” in most of the diseases. A “high” quality of evidence was found in studies of PD and stroke populations, that is the populations in which most studies assessing the construct validity of the BESTest were conducted, “moderate” in mixed neurological diseases and traumatic brain injury, “low” and “very low” in Alzheimer disease, multiple sclerosis, cerebral palsy, and CSM, mainly due to the paucity of studies and their related small sample sizes.

### Responsiveness

For assessing responsiveness, in addition to the studies clearly assessing this psychometric property, those studies in which the exercise training was known to have an effect on the balance construct (ie, balance exercise training) were considered. Therefore, 6 studies were identified assessing the responsiveness of the BESTest in PD and subacute stroke^29^^,^^33^^,^^35^^,^^41^ (Table 2). Methodological quality of 2 studies^27^^,^^31^ was rated as “excellent,” 1 study^29^ was rated as “good,” 2 studies^33^^,^^35^ as “fair” due to the inadequate description of the subgroups, while 1 study^41^ was rated as “poor” since the rehabilitative intervention was not described. Sensitivity to change was assessed comparing data at baseline and at the end of treatment, which ranged between 13 and 84 days. Measures of change (ie, effect size or SRM) were assessed in each study, or calculated from the review team from the published data, while the AUC using the BBS or the Global Rating of Change score as anchor in only 3 studies. Results of 1 study^29^ was rated considering the hypotheses reported in the paper, while the other results were assessed through the preformulated hypotheses. Based on the data reported in the studies, most of the results were in accordance with the hypotheses (92%). Therefore, overall evidence on responsiveness was rated as “sufficient,” with a “high” quality (Table 3). Considering the study groups separately, the responsiveness’ quality of evidence was “high” in PD only.

## DISCUSSION

To the author’s knowledge, this is the first systematic review which aimed at synthesizing evidence regarding the psychometric properties of the BESTest across neurological populations. However, previous systematic reviews have assessed the measurement quality of 2 short form of the BESTest, the Mini-BESTest and the Brief-BESTest.^60^^,^^61^ Results of the current, systematic review of the BESTest in heterogeneous neurological populations showed that 6 measurement properties were rated high (content and construct validity, internal consistency, reliability, measurement error and responsiveness). However, structural validity had low quality evidence and cross-cultural validity has not been studied yet. These results suggest that clinicians/researchers can be confident that the scores of the BESTest have a high degree of validity and reliability and are likely to measure the constructs they are designed to measure, but some properties still need to be clarified.

The original BESTest development paper^9^ provided a clear description of the construct to be measured, based on the 6 systems underlying postural control.^3^ The target population was clearly described and consisted of people with a variety of balance dysfunctions. Modifications to the newly developed BESTest were done thanks to the feedback about clarity, sensitivity and practicality of items given by thousands of experienced physical therapy clinicians. Due to that accuracy, high evidence for sufficient quality of BESTest development and content validity was obtained. Despite generally positive findings, a content validity issue was identified with the practicality of item #27 (“TUG With Dual Task”), since instruction of this item asks patients to “count backwards from a number between 90 and 100 by 3s.” This task could be quite challenging for some individuals who have aphasia, cognitive deficits, poor concentration or who are illiterate. An appropriate alternative dual-motor task (ie, walk with a cup of water) has been proposed for those patients having difficulty in performing the cognitive task of item #27 and adopted for the Mini-BESTest, the short form of the BESTest including the same item.^62^^,^^63^

Only 3 studies^26^^,^^36^^,^^52^ of limited quality investigated the Structural Validity measurement property. However, compared to the short version scales derived from the BESTest (Mini-BESTest, Brief-BESTest, S-BESTest) or other scales commonly used for measuring balance (ie, BBS), the BESTest aims to measure several different aspects of balance. The few studies conducted so far suggest that the BESTest scale actually measures 4 underlying systems of postural control (ie, “Postural Responses,” “Sensory Orientation,” “Stability in Gait,” and “Stability Limits”) with respect to the 6 systems originally hypothesized by the developers of the scale.^9^^,^^36^^,^^52^ Nevertheless, the BESTest scale cannot be considered unidimensional since there was more evidence in favour, than against, the multidimensional nature of this scale. Future studies should determine the exact number of underlying postural systems the BESTest is able to measure across different neurological populations, possibly performing a CFA with an adequate sample size (≥300 participants).

Although the evidence on internal consistency was rated as excellent for the BESTest, it needs to be noted that unidimensionality of the subscales is a prerequisite for interpreting internal consistency. As evidence for the structural validity of the BESTest is limited, our rating of the evidence on internal consistency is intended as an interpretation for the 3 postural control systems that have consistently been found as being unidimensional: section IV (“Postural Responses”), section V (“Sensory Orientation”) and section VI (“Stability in Gait”).

Measurement error was mainly assessed with the MDC_95_. While the values ranged between 3 and 22, most of the results were between 3 and 9, that is lower than the MCID values assessed in the literature. This finding denotes that when a BESTest change is greater than the MDC_95_ assessed for that specific neurological condition, clinicians can be confident that this change is real and is not due to random variability.

Criterion validity cannot be assessed since there is no gold standard for measuring the multidimensional “balance” construct. While the BESTest is the gold standard for it’s short forms (Mini-BESTest and Brief-BESTest), the opposite is not true. Although construct validity was the measurement property assessed in the largest number of studies and in all populations, current studies did not state their *a priori* hypotheses regarding convergent or discriminative validity, with the exception of a spinocerebellar ataxia study. The BESTest demonstrated high associations with balance scales measuring similar constructs, such as it’s short forms and the BBS. Similar high associations were also found in previous systematic reviews assessing the measurement properties of the Brief-BESTest and the Mini-BESTest.^60^^,^^61^ The BESTest also showed moderate-to-high correlations with scales assessing related, but dissimilar, constructs, such as the Activities of Balance Confidence patient-reported scale and measures related to gait. Results of the present review also strongly support also the discriminative, or known-group validity, of the BESTest for its ability to identify patients based on their history of falls or physical performance, and in predicting patients with a falling risk.

The BESTest total scores demonstrated excellent interrater, intrarater, and test–retest reliability. Test–retest reliability of subsections also showed “good-to-excellent” reliability, except for Section II (“Stability limits”) that demonstrated only moderate reliability. Interrater reliability was found to be “excellent” for the total score and “good” for the subsections. In general, values of interrater reliability were larger than those of test–retest reliability. These results are in accordance with the analysis of individual BESTest items conducted by Horak et al^9^ during the development of BESTest. Items based on stopwatch time, such as those collected in section VI (“Sensory Orientation”), tended to show the highest concordance, whereas judgments of alignment, sitting limits of stability and verticality tended to show the lowest concordance. Previous studies^64^^,^^65^ analysing the reliability of the Mini-BESTest found low concordance values for items that covered the section “Reactive Postural Control.”^66^ The authors of these studies proposed that the suboptimal agreement in these item scores might be due to inconsistent resistive forces applied by the examiners, which might cause variable postural responses. This hypothesis is not supported by our results that showed good reliability values of Section IV (“Postural Responses”) for both the test–retest and the interrater reliability. Rather, the difference in reliability values more likely depends on the 3-point scale of the Mini-BESTest versus the 4-point scale of the BESTest. Thus, the reliability findings from the BESTest may not be generalized to the short versions of the scale.

Compared to reliability and validity domains, responsiveness was the measure less frequently assessed. According to the COSMIN taxonomy, responsiveness of a test should be determined by comparing at least 2 measurements over time with the intervention known to have an effect on the contruct measures (ie, balance) or the score changes should be correlated with changes in other variables (ie, other instruments, demographic or clinical).^67^^,^^68^ Most of the studies using the BESTest as an outcome measure^69–71^ did not include changes in other measures.^68^ The methodological quality of the 4 included studies assessing responsiveness was not good. Nevertheless, the overall responsiveness of the BESTest was rated as sufficient since >75% of the hypotheses were confirmed. Concerning the external responsiveness, the AUC were mainly >0.70,^15^ with a reported cut-off, corresponding to the MCID, of about 8% to 17% of the total score of the BESTest. These results are consistent with the literature, based on which the MCID should be of about 10% to 15% of the total score of a scale.^72^ Therefore, future studies are warranted to establish MCIDs for the BESTest total score and its subsections in various populations other than stroke.

### Limitations

One limitation of this review was the inclusion of a heterogeneous sample of PwNC. It is known that PwNC manifest a wide variety of balance dysfunctions that are caused by different pathophysiological mechanisms.^73^ Due to the small number of studies found for each neurological condition, however, it was not possible to focus on a single neurological population. However, we do include a summary of the overall rating and quality of evidence for each measurement property of the BESTest in each neurological population (see Table 3). Another potential limitation is that studies not published in English or Italian, and those not peer-reviewed were not included in this review.

### Clinical Implications

This review establishes the groundwork for the use of the BESTest in PwNC, given its strong levels of reliability, validity, and responsiveness (Table 3). The only aspects that have not yet been adequately studied are cross-cultural validity and criterion validity. Cross-cultural validity studies are needed since the test has been translated and back-translated into many other languages but studies comparing results across cultures have not been reported. Criterion validity is impossible since, validity cannot be measured in the absence of a gold standard.^18^ It is worth noting that the quality of evidence is high for PD and stroke but not other diseases, mainly due to small sample sizes.^18^ Nevertheless, the psychometric properties obtained by the BESTest, across a heterogeneous population of PwNC, are at least comparable to other well-established balance scales.^10^

There exist over 66 standardized balance scales. The BESTest is the only scale designed to assess all 6 components of postural control outlined in the Systems Framework for Postural Control.^9^^,^^10^ The BESTest is particularly suitable for patients with PD and stroke, where the BESTest already demonstrates high levels of methodological quality for both clinical and research use. However, if therapists need a shorter balance test with similar, good psychometric properties, they should consider shorter scales such as the Mini-BESTest. Future, well-designed large-scale clinical studies should focus on the structural validity of the BESTest, investigating the unidimensionality of each subscore with an adequate sample size, its responsiveness in populations other than stroke and PD, and its cross-cultural validity.

## Supplementary Material

2023-0607_R2_Supplementary_Material_rev_pzae178

## Data Availability

Data sharing is not applicable to this article as no new data were created or analyzed in this study.

## SYSTEMATIC REVIEW REGISTRATION

This review was registered on PROSPERO (No. CRD4202340773).
