# Supplementary material for: Measurement Properties of the BESTest Scale in People With Neurological Conditions: A Systematic Review With Meta-Analysis
Source: Phys Ther. 2024 Dec 12;105(3):pzae178. doi: 10.1093/ptj/pzae178 (PMC11890282; doi:10.1093/ptj/pzae178)

Supplementary Table 1. Search Strategy

Step	Embase
1	Bestest
2	('neurological disease'/exp OR 'neurological disease')
3	S1 AND S2
**Step**	**Medline**
1	(''Bestest"[All Fields])
2	("nervous system diseases"[MeSH Terms] OR ("nervous"[All Fields] AND "system"[All Fields] AND "diseases"[All Fields]) OR "nervous system diseases"[All Fields] OR ("neurological"[All Fields] AND "disease"[All Fields]) OR "neurological disease"[All Fields])
3	S1 AND S2
**Step**	**PEDro**
1	BESTest*
**Step**	**Scopus**
1	Bestest
2	Balance
3	Rehabilitation
4	(''Neurological disease'')
5	SUBJAREA(medi) AND SUBJAREA(heal) AND SUBJAREA(neur) AND SUBJAREA(nurs)
6	S1 AND S2 AND S3 AND S4 AND S5
**Step**	**Science Direct**
1	Bestest

Note: the keywords and medical subject headings were modified slightly to optimize the searches in various databases.

Supplementary Table 2. Specific hypotheses formulated by the review team for the BESTest scale for assessing construct validity and responsiveness.[a]

**CONSTRUCT VALIDITY**	
Comparison with other outcome measurement instruments (convergent validity)	
1	In all neurological populations, large correlations (≥ 0.50) would be observed between the BESTest scale[b] and comparator instruments measuring *similar* constructs (e.g., Mini-BESTest, Brief-BESTest, BBS).
2	In all neurological populations, moderate to large correlations (≥ 0.30 and ≤ 0.70) would be observed between the BESTest scale[b] and instruments measuring *related but dissimilar* constructs (e.g., ABC, gait speed)
3	In all neurological populations, weak correlations (≤ 0.30) would be observed between the BESTest scale[b] and instruments or characteristics measuring *unrelated* constructs (e.g., PASE).
4	Correlations defined under hypotheses 1, 2 and 3 should differ by a minimum of 0.10.
Comparison between subgroups (Discriminative or known-groups validity)	
5	The BESTest score[b] should be able to distinguish between subgroups of subjects based on their history of fall, with ES ≥ 0.70 OR AUC ≥ 0.70.
6	The BESTest score[b] should be able to distinguish between subgroups of subjects based on their risk of fall, with ES ≥ 0.70 OR AUC ≥ 0.70.
7	The BESTest score[b] should be able to distinguish between subgroups of subjects based on their physical performance, with ES ≥ 0.70 OR AUC ≥ 0.70.
**RESPONSIVENESS**	
Before and After Intervention:	
1	In response to a rehabilitative intervention, a moderate to large improvement in BESTest scores[b] (i.e., ES ≥ 0.50[c]) would be found in the intervention group, but not in the control group (if present).
2	In response to a rehabilitative intervention, at least a moderate difference in post-intervention BESTest scores[b] (or change scores) would be found between the intervention and control groups, with the intervention group showing greater improvement (i.e., ES ≥ 0.50[c]).
Comparison between subgroups	
3	Ability of the BESTest to discriminate between improved and not-improved (or independent and not-independent) groups (AUC ≥ 0.70) following rehabilitative intervention.
Comparison with other outcome measurement instruments	
4	Changes in BESTest scores[b] would have moderate to large correlations (i.e., ≥ 0.30 and ≤ 0.70) with changes in instruments measuring similar constructs (e.g., Mini-BESTest, Brief-BESTest, BBS).
5	Changes in BESTest scores[b] would have small to moderate correlations (i.e., ≥ 0.30 and < 0.5) with instruments assessing perceived change (e.g., GRC).

[a] ABC, Activities-specific Balance Confidence scale; AUC: area under the curve; BBS: Berg Balance Scale; ES: effect size; GRC: global rating of change; PASE: Physical Activity Scale for the Elderly.

[b] If the BESTest total score was not reported, the subscores were considered.

[c] If ES was not reported, but data for its calculation were reported, then it was calculated by the review team.

Supplementary Table 3. Methodological quality and results of studies on measurement properties of validity domain.[a]

Population and Study	Structural validity	COSMIN Score / Quality Score	Construct validity *Convergent validity*	Construct validity *Discriminative or known-groups validity*	COSMIN Score / Quality Score
**Alzheimer's Disease**					
Tueth et al[49] (2021)	NA	NA	NA	**Fallers vs non-fallers (12-month falling risk)**: total score % (SD) = 58.2 (9.6) vs 73.9 (7.9), p = 0.001; ES = 1.85. Data available also for each section.	Excellent (+) 1/1 hypothesis confirmed
**Cerebral Palsy**					
Morgan et al[46] (2016)	NA	NA	**Preferable walk**: r = 0.57 with speed; r = 0.26 with stride length; r = -0.08 with step width; r = 0.28 with cadence; r = -0.61 with double support time; r = -0.47 with double support time variability. **Fast walk**: r = 0.65 with speed; r = 0.27 with stride length; r = -0.02 with step width; r = 0.49 with cadence; r = -0.67 with double support time; r = -0.50 with double support time variability. Correlations available also for each section.	**Fallers vs non-fallers**: total score % (SD) = 46.9 (15.3) vs 57.9 (29.6), p = 0.34. Data available also for each section.	Poor (-) 0/2 hypotheses confirmed

Opheim et al[47] (2012)	NA	NA	r = 0.57 (p = 0.02) with ABC. Correlations available also for each section.	NA	Excellent (+) 1/1 hypothesis confirmed
**Cervical Spondylotic Myelopathy**					
Chiu and Pang[48] (2017)	NA	NA	r = 0.89-0.99 with BBS/Mini-BESTest/Brief-BESTest; r = 0.81-0.84 (p < 0.001) with mJOA; r = 0.09-0.37 with AMT.	**Fallers vs non-fallers:** AUC (95% CI) = 0.89 (0.79-0.95), cutoff point = 65, sensitivity (95% CI) = 80.6 (62.5-92.5), specificity (95% CI) = 90.2 (76.9-97.3), LR+ = 8.27, LR- = 0.21. **With vs without mobility aids:** AUC (95% CI) = 0.95 (0.87-0.99), cutoff point = ≤71, sensitivity (95% CI) = 93.3 (77.9-99.2), specificity (95% CI) = 86.4 (71.2-95.5), LR+ = 6.91, LR- = 0.08.	Fair (+) 5/6 hypotheses confirmed
**Mixed neurological diseases**					
Franchignoni et al[52] (2010)	**CFA:** Unidimensional model: NNFI = 0.91, CFI = 0.91, RMSEA = 0.12, SRMR = 0.15.	Poor (-)	NA	NA	NA
Hamre et al[53] (2017)	NA	NA	rs = -0.51 (p = 0.01) with FES-I	NA	Excellent (+) 1/1 hypothesis confirmed

Horak et al[9] (2009)	NA	NA	r = 0.69 ($p < 0.05$) with ABC Scale. Subscores correlation range = 0.41-0.78.	NA	Excellent (+) 1/1 hypothesis confirmed
Padgett et al[54] (2012)	NA	NA	NA	**Fallers vs non-fallers:** total score % (95% CI) = 77 (66-87) vs 96 (94-98).	Fair (+) 1/1 hypothesis confirmed
**Multiple Sclerosis**					
Jacobs and Kasser[42] (2012)	NA	NA	r = 0.85 ($p < 0.0005$) with EDSS.	NA	Fair (-) 0/1 hypothesis confirmed
Mitchell et al[43] (2018)	NA	NA	r = 0.94 (p = 0.01) with BBS; r = -0.88 (p = 0.01) with EDSS.	**Fallers vs non-fallers:** AUC = 0.76, cutoff point = 81%, sensitivity = 0.89, specificity = 0.64.	Fair (-) 2/3 hypotheses confirmed
Potter et al[44] (2018)	NA	NA	r = -0.69 with Self-perceived Disability Level. r = -0.61 with Fall Frequency. Correlations available also for each section.	NA	Poor (+) 1/1 hypothesis confirmed
**Parkinson's Disease**					
Duncan et al[20] (2012)	NA	NA	NA	**Fallers vs non-fallers (falling risk at 6 months):** AUC (95% CI) = 0.89 (0.74-0.95), cutoff point = 69%, sensitivity = 0.93, specificity = 0.84, LR+ (95% CI) = 5.81 (3.69-9.14), LR- (95% CI) = 0.08 (0.04-0.17). **Fallers vs non-fallers (falling risk at 12 months):** AUC (95% CI) = 0.68 (0.45-0.83), cutoff point = 69%, sensitivity = 0.46, specificity = 0.74, LR+ (95% CI) = 1.77 (1.19-2.62), LR-	Fair (-) 1/2 hypotheses confirmed

				(95% CI) = 0.73 (0.59-0.91).	
Duncan et al[21] (2012)	NA	NA	r = 0.95 (p = 0.01) with Brief-BESTest. Correlations available also for each section.	**Fallers vs non-fallers:** total score % (SD) = 57.2 (15.3) vs 76.4 (13.6), p = 0.34. AUC (95% CI) = 0.84 (0.75-0.93), cutoff point = 69%, sensitivity = 0.84, specificity = 0.76, LR+ (95% CI) = 3.49 (2.11-5.77), LR- (95% CI) = 0.21 (0.09-0.52). **Fallers vs non-fallers (falling risk at 6 months):** AUC (95% CI) = 0.89 (0.74-0.95), cutoff point = 69%, sensitivity = 0.93, specificity = 0.84, LR+ (95% CI) = 5.81 (3.69-9.14), LR- (95% CI) = 0.08 (0.04-0.17). **Fallers vs non-fallers (falling risk at 12 months):** AUC (95% CI) = 0.68 (0.45-0.83), cutoff point = 69%, sensitivity = 0.46, specificity = 0.74, LR+ (95% CI) = 1.77 (1.19-2.62), LR- (95% CI) = 0.73 (0.59-0.91).	Good (+) 3/4 hypotheses confirmed

Duncan et al[22] (2015)	NA	NA	**Correlation at 6 months:** r = -0.39 with MDS-UPDRS-III; r = -0.28 with H&Y; Freezer status r = -0.52; r = 0.23 with PASE; r = -0.30 with fall history; r = -0.17 with age; r = 0.06 with gender. **Correlation at 12 months:** r = -0.42 with MDS-UPDRS-III; r = -0.18 with H&Y; Freezer status r = -0.39; r = 0.11 with PASE; r = -0.14 fall history; r = -0.18 with age; r = -0.05 with gender.	NA	Excellent (+) 3/3 hypotheses confirmed
Duncan et al[23] (2015)	NA	NA	NA	**With FOG vs without FOG**: total score % (SD) = 59.7 (15.5) vs 77.3 (13.5), p = 0.008; ES = 1.21. Data available also for each section.	Good (+) 1/1 hypothesis confirmed
Leddy et al[24] 2011	NA	NA	r = 0.87 with BBS; r = -0.74 with H&Y; r = -0.76 with MDS-UPDRS III; r = -0.78 with MDS-UPDRS tot; r = 0.76 with ABC; r = 0.88 with FGA.	**Fallers vs non-fallers:** AUC (95% CI) = 0.84 (0.75-0.93), cutoff point = 84%, sensitivity = 1.00, specificity = 0.39, LR+ (95% CI) = 1.64 (1.32-2.02), LR- (95% CI) = 0.00 (unable to calculate).	Good (-) 2/4 hypotheses confirmed
Leddy et al[25] 2011	NA	NA	r = 0.96 with Mini-BESTest.	**Fallers vs non-fallers:** AUC (95% CI) = 0.84 (0.75-0.93), cutoff point = 69%, sensitivity = 0.84, specificity = 0.76, LR+ (95% CI) = 3.49 (2.11-5.77), LR-	Excellent (+) 2/2 hypotheses confirmed

				(95% CI) = 0.21 (0.09-0.52).	
Maia et al[26] (2013)	**Rasch analysis:** individual separation index = 4.19; item separation index = 5.36; maximum infit: MnSq = 1.62, t = 3.1; maximum outfit: MnSq = 1.89, t = 3.8; minimum infit: MnSq = 0.45, t = -4.2; minimum outfit: MnSq = 0.53, t = -3.1.	Poor (?)	NA	NA	NA
Santos et al[28] (2023)	NA	NA	r = -0.61 (p < 0.001) with H&Y.	H&Y I vs H&Y II vs H&Y III: total score (SD) = 89.72 (4.96) vs 82.69 (7.35) vs 73.85 (9.89), p < 0.001; ES = 0.99.	Excellent (+)<br>2/2 hypotheses confirmed
Warlop et al[30] (2016)	NA	NA	r = 0.64 with the ABC; r = 0.40 with gait speed; r = -0.38 with stride duration CV; r = -0.90 with the H&Y; r = -0.73 with the MDS-UPDRS-III; r = -0.05 with age.	NA	Excellent (+)<br>3/3 hypotheses confirmed
**STROKE**					
Subacute stroke					
Chinsongkram et al[32] (2014)	NA	NA	r = 0.96 with the BBS; r = 0.96 with the Mini-BESTest; r = 0.96 with the PASS; r = 0.91 with the CB&M.	**High vs low performer**: AUC (95% CI) = 0.88 (0.80-0.95), cutoff point = 49%, sensitivity (95% CI) = 71.4 (54.7-83.7), specificity (95%CI) = 91.4 (76.7-97.7), LR+ (95% CI) = 8.33 (2.77-25.09), LR- (95% CI) = 0.31 (0.18-0.53), post-test accuracy = 0.81%.	Excellent (+)<br>2/2 hypotheses confirmed

Winairuk et al[41] (2019)	NA	NA	r = 0.96 with the BBS.	NA	Excellent (+)<br>1/1 hypothesis confirmed
Chronic Stroke					
Miyata et al[36] (2018)	**CFA:**<br>Unidimensional model: χ^2/df = 2.2, RMSEA = 0.09, CFI = 0.73, TLI = 0.71;<br>6-factor model: χ^2/df = 1.8, RMSEA = 0.07, CFI = 0.83, TLI = 0.82;<br>4-factor model: χ^2/df = 1.8, RMSEA = 0.08, CFI = 0.90, TLI = 0.89.	Poor (-)	Correlations of the four subscores of the 25-item model with the BRS: *factor 1:* r = 0.50; *factor 2:* r = 0.36; *factor 3:* r = 0.24; *factor 4:* r = 0.30.<br>Correlations of the four subscores of the 25-item model with the 10mWT: *factor 1*: r = 0.79; *factor 2*: r = 0.66; *factor 3*: r = 0.58; *factor 4*: r = 0.42.	NA	Good (+)<br>1/1 hypothesis confirmed
Miyata et al[37] (2018)	NA	NA	NA	**Fallers vs non-fallers (6-month falling risk):**<br>*section I*: AUC (95% CI) = 0.82 (0.61-1.00), cutoff point = 76.7%, sensitivity = 0.86, specificity = 0.60, LR+ = 2.14, LR- = 0.30;<br>*section II*: AUC (95% CI) = 0.61 (0.21-1.00), cutoff point = 78.6%, sensitivity = 0.93, specificity = 0.60, LR+ = 2.32, LR- = 0.35;<br>*section III*: AUC (95% CI) = 0.83 (0.56-1.00), cutoff point = 63.9%, sensitivity = 0.79, specificity = 0.80, LR+ = 3.92, LR- = -0.02;<br>*section IV*: AUC (95% CI) = 0.56 (0.22-0.90), cutoff point = 69.5%; sensitivity = 0.71, specificity = 0.60, LR+ = 1.79, LR- = 0.16;<br>*section V*: AUC (95% CI) =	Excellent (+)<br>1/1 hypothesis confirmed

0.84 (0.66-1.00), cutoff point = 83.3%, sensitivity = 0.64, specificity = 1.00, LR+ = /, LR- = -0.56; *section VI*: AUC (95% CI) = 0.81 (0.54-1.00), cutoff point = 69.1%, sensitivity = 0.93, specificity = 0.80, LR+ = 4.64, LR- = 0.13.

Miyata et al[38] (2021)	NA	NA	NA	**Household vs limited community ambulators**: *section I*: AUC (95% CI) = 0.88 (0.78-0.98), cutoff point = 50.0%, sensitivity = 93.1, specificity = 76.5, LR+ = 87.1, LR- = 86.7; *section II*: AUC (95% CI) = 0.77 (0.62-0.93), cutoff point = 78.6%, sensitivity = 82.8, specificity = 70.6, LR+ = 82.8, LR- = 70.6; *section III*: AUC (95% CI) = 0.84 (0.71-0.96), cutoff point = 36.1%, sensitivity = 100.0, specificity = 52.9, LR+ = 78.4, LR- = 100.0; *section IV*: AUC (95% CI) = 0.77 (0.61-0.93), cutoff point = 41.7%, sensitivity = 93.1, specificity = 58.8, LR+ = 79.4, LR- = 83.3; *section V*: AUC (95% CI) = 0.81 (0.67-0.95), cutoff point = 63.4%, sensitivity = 93.1, specificity = 64.7, LR+ = 81.8, LR- = 84.6; *section VI*: AUC (95% CI) = 0.90 (0.80-0.99), cutoff point = 50.0%, sensitivity = 82.8, specificity = 82.4, LR+ = 88.9, LR- = 73.7. **Limited community vs unlimited community ambulators:** *section I*: AUC (95% CI) = 0.82 (0.72-0.92), cutoff point = 83.4%, sensitivity = 73.2, specificity = 82.8, LR+	Good (+)<br>1/1 hypothesis confirmed

				= 85.7, LR- = 68.6; *section II*: AUC (95% CI) = 0.67 (0.54-0.79), cutoff point = 92.9%, sensitivity = 39.0, specificity = 86.2, LR+ = 80.0, LR- = 50.0; *section III*: AUC (95% CI) = 0.83 (0.74-0.93), cutoff point = 75.0%, sensitivity = 63.4, specificity = 93.1, LR+ = 92.9, LR- = 64.3; *section IV*: AUC (95% CI) = 0.81 (0.71-0.92), cutoff point = 80.6%, sensitivity = 68.3, specificity = 82.8, LR+ = 84.8, LR- = 64.9; *section V*: AUC (95% CI) = 0.68 (0.55-0.81), cutoff point = 90.0%, sensitivity = 61.0, specificity = 72.4, LR+ = 75.8, LR- = 56.8; *section VI*: AUC (95% CI) = 0.91 (0.84-0.97), cutoff point = 73.8%, sensitivity = 80.5, specificity = 89.3, LR+ = 91.7, LR- = 76.5.	
Rodrigues et al[39] (2014)	NA	NA	r = 0.78 with BBS; r = 0.59 with ABC. Correlations available also for each section.	NA	Excellent (+)<br>3/3 hypotheses confirmed
Sahin et al[40] (2019)	NA	NA	r = 0.91 with BBS; r = 0.89 (p < 0.001) with ABC; r = 0.62 with postural stability; r = 0.60 with limits of stability; r = 0.43, r = 0.33, r = 0.38, r = 0.20 with the four conditions of the MSOT.	**Fallers vs non-fallers:** AUC (95% CI) = 0.84 (0.73-0.96), cutoff point = 69, sensitivity = 0.75, specificity = 0.85, LR+ (95% CI) = 3.38 (2.5-4.5), LR- (95% CI) = 0.21 (0.07-0.6).	Good (+)<br>4/4 hypotheses confirmed

			Correlations available also for each section.	Data available also for each section.	
**Traumatic Brain Injury**					
Hays et al[51] (2019)	NA	NA	r = 0.46 with the ABC; r = 0.86 with CB&M. Correlations with CB&M available also for each section.	NA	Excellent (+)<br>3/3 hypotheses confirmed

[a] ABC = Activities-specific balance confidence scale; AMT = Abbreviated Mental Test; AUC = Area under the Receiver Operating Characteristic curve; BBS = Berg Balance Scale; CB&M = Community Balance and Mobility; CFA = confirmatory factor analysis; CFI = Comparative fit index; df = degree of freedom; EDSS = Expanded Disability Status Scale; ES = effect size; FES-I = Falls Efficacy Scale International; FGA = Functional Gait Assessment; df = degree of freedom; H&Y = Hoehn and Yahr; ICC = intra-class correlation coefficients; LR+ = positive likelihood ratio; LR- = negative likelihood ratio; MDS-UPDRS = Movement Disorder Society-Sponsored Revision of the Unified Parkinson's Disease Rating Scale; mJOA = modified Japanese Orthopaedic Association; MnSq = mean square; MSOT = Modified Sensory Organization Test; NA = not available; NNFI = non-normed fit index; PASE = Physical Activity Scale for the Elderly; PASS = Postural Assessment Scale for Stroke Patients; RMSEA = Root mean square error of approximation; SRMR = standardized root mean square residual; TLI = Tucker-Lewis index; χ^2 = chi-square.

Supplementary Table 4. Methodological quality and results of studies on measurement properties of reliability domain.[a]

Population and Study	Internal Consistency	COSMIN Score / Quality Score	Reliability Interrater	Reliability Intra-rater	Reliability Test-retest	COSMIN Score / Quality Score	Measurement error	COSMIN Score / Quality Score
**Cerebral Palsy**								
Levin et al[45] (2019)	NA	NA	NA	NA	ICC(2,1) (95% CI): *total* = 0.99 (0.96-0.99), *section I* = 0.97 (0.93-0.99), *section II* = 0.88 (0.73-0.95), *section III* = 0.97 (0.92-0.99), *section IV* = 0.93 (0.84-0.97), *section V* = 0.89 (0.75-0.96), *section VI* = 0.98 (0.95-0.99).	Fair (+)	SEM based on test-retest: *total* = 1.76, *section I* = 3.13, *section II* = 3.23, *section III* = 3.83, *section IV* = 6.87, *section V* = 6.56, *section VI* = 3.22. MDC_{95}: *total* = 4.9, *section I* = 8.7, *section II* = 9.0, *section III* = 10.6, *section IV* = 19.0, *section V* = 18.2, *section VI* = 8.9.	Fair (+)
**Cervical Spondylotic Myelopathy**								
Chiu and Pang[48] (2017)	Cronbach's Alpha: *section I* = 0.85, *section II* = 0.85, *section III* = 0.91, *section IV* = 0.98, *section V* = 0.85, *section VI* = 0.96.	Excellent (+)	ICC(2,1) (95% CI): *total* = 0.99 (0.98-1.00).	NA	ICC(2,1) (95% CI): *total* = 0.99 (0.99-1.00).	Good (+)	SEM based on test-retest: *total* = 1.15 (0.92-1.53), MDC_{95}: *total* = 3.19.	Good (+)
**Mixed neurological diseases**								

Hamre et al[53] (2017)	NA	NA	ICC(3,1) (95% CI):<br>*total* = 0.99 (0.97–0.99),<br>*section I* = 0.87 (0.77–0.93),<br>*section II* = 0.95 (0.90–0.97),<br>*section III* = 0.97 (0.94–0.98),<br>*section IV* = 0.96 (0.93–0.98),<br>*section V* = 0.99 (0.99–0.99),<br>*section VI* = 0.94 (0.89–0.97).	NA	ICC(3,1) (95% CI):<br>*total* = 0.93 (0.87–0.96),<br>*section I* = 0.68 (0.48–0.82),<br>*section II* = 0.53 (0.27–0.72),<br>*section III* = 0.83 (0.71–0.91),<br>*section IV* = 0.68 (0.47–0.81),<br>*section V* = 0.87 (0.77–0.93),<br>*section VI* = 0.85 (0.73–0.92).	Excellent (+)	SEM based on interrater:<br>*total* = 1.79,<br>*section I* = 0.93,<br>*section II* = 1.39,<br>*section III* = 0.56,<br>*section IV* = 0.71,<br>*section V* = 0.22,<br>*section VI* = 1.01;<br>SDC_{95}:<br>*total* = 4.96,<br>*section I* = 2.57,<br>*section II* = 3.85,<br>*section III* = 1.55,<br>*section IV* = 1.98,<br>*section V* = 0.60,<br>*section VI* = 2.79.	Excellent (+)

Horak et al[9] (2009)	NA	NA	ICC(2,1) (95% CI): *total* = 0.91 (0.83-0.97), *section I* = 0.80 (0.63-0.93), *section II* = 0.79 (0.63-0.92), *section III* = 0.92 (0.85-0.97), *section IV* = 0.92 (0.85-0.97), *section V* = 0.96 (0.92-0.99), *section VI* = 0.88 (0.76-0.96); Kendall coefficient (95% CI): *section I* = 0.79 (0.73-0.85), *section II* = 0.86 (0.84-0.88), *section III* = 0.92 (0.91-0.93), *section IV* = 0.91 (0.90-0.92), *section V* = 0.95 (0.95-0.95), *section VI* = 0.93 (0.90-0.96).	NA	NA	Excellent (+)	NA	NA
Padgett et al[54] (2012)	Cronbach's alpha: *section I* = 0.84, *section II* = 0.62, *section III* = 0.87, *section IV* = 0.86, *section V* = 0.81, *section VI* = 0.92.	Excellent (+)	ICC (95% CI): *total* = 0.99 (0.96–0.99).	NA	NA	Good (+)	NA	NA

**Multiple Sclerosis**								
Mitchell et al[43] (2018)	NA	NA	NA	NA	ICC(3,1):<br>*total* = 0.98.	Fair (+)	SEM based on test-retest:<br>*total* = 4.16;<br>MDC_{95}:<br>*total* = 11.5%.	Fair (-)
Potter et al[44] (2018)	Cronbach's Alpha:<br>*total* = 0.97,<br>*section I* = 0.92,<br>*section II* = 0.83,<br>*section III* = 0.91,<br>*section IV* = 0.85,<br>*section V* = 0.79,<br>*section VI* = 0.96.	Excellent (+)	NA	NA	ICC (95% CI):<br>*total* = 0.94 (0.86-0.98),<br>*section I* = 0.85 (0.67-0.94),<br>*section II* = 0.71 (0.41-0.87),<br>*section III* = 0.84 (0.64-0.93),<br>*section IV* = 0.74 (0.45-0.88),<br>*section V* = 0.66 (0.32-0.85),<br>*section VI* = 0.93 (0.84-0.97),<br>with Bland Altman Plots.	Good (+)	MDC_{95} based on test-retest:<br>*total* = 9.47,<br>*section I* = 2.26,<br>*section II* = 2.25,<br>*section III* = 2.62,<br>*section IV* = 4.49,<br>*section V* = 2.46,<br>*section VI* = 4.58.	Good (+)
**Parkinson's Disease**								
Leddy et al[25] (2011)	NA	NA	ICC(2,1) (95% CI):<br>*total* = 0.96 (0.89-0.99),<br>*section I* = 0.81 (0.61-0.92),<br>*section II* = 0.79 (0.58-0.92),<br>*section III* = 0.91 (0.81-0.97),<br>*section IV* = 0.91 (0.81-0.97),	NA	ICC(2,1) (95% CI):<br>*total* = 0.88 (0.72-0.95),<br>*section I* = 0.69 (0.41-0.85),<br>*section II* = 0.63 (0.31-0.82),<br>*section III* = 0.83 (0.45-0.94),<br>*section IV* = 0.87 (0.68-0.95),	Fair (+)	NA	NA

			section V = 0.96 (0.91-0.99), *section VI* = 0.86 (0.62-0.95).		*section V* = 0.72 (0.44-0.87), *section VI* = 0.72 (0.45-0.87).			
Maia et al[26] (2013)	NA	NA	NA	NA	ICC: *total* = 0.92.	Fair (+)	NA	NA
Silva-Batista et al[29] (2018)	NA	NA	NA	NA	NA	NA	MDC_{95}: total = 6.4%.	Poor (+)
**Spinocerebellar ataxia**								
Kondo et al[50] (2020)	NA	NA	NA	NA	ICC(1,1): *total* = 0.92, *section I* = 0.70, *section II* = 0.77, *section III* = 0.85, *section IV* = 0.72, *section V* = 0.90, *section VI* = 0.96.	Fair (+)	SEM based on test-retest: *total* = 3.2, *section I* = 0.8, *section II* = 0.8, *section III* = 0.9, *section IV* = 2.5, *section V* = 0.7, *section VI* = 1.0; MDC_{95}: *total* = 8.7, *section I* = 2.3, *section II* = 2.2, *section III* = 2.6, *section IV* = 6.9, *section V* = 2.0, *section VI* = 2.8.	Fair (+)
**STROKE**								
Subacute stroke								

Chinsongkram et al[32] (2014)	NA	NA	ICC (95% CI): *total* = 0.99 (0.98-0.99); subsections ranging from 0.87 (0.72-0.96) to 0.98 (0.95-0.99).	ICC (95% CI): *total* = 0.99 (0.99-1.00); subsections ranging from 0.95 (0.90-0.98) to 0.99 (0.98-0.99).	NA	Fair (+)	NA	NA
Winairuk et al[41] (2019)	NA	NA	NA	NA	NA	NA	MDC_{95} (0-2weeks): *total* = 9.47; MDC_{95} (2-4 weeks): *total* = 7.29.	Poor (+)
Chronic Stroke								
Dadbakhsh et al[34] (2023)	NA	NA	NA	NA	ICC(2,1) (95% CI): *total* = 0.88 (0.73–0.95), *section I* = 0.72 (0.28–0.89), *section II* = 0.55 (0.12–0.80), *section III* = 0.79 (0.53–0.91), *section IV* = 0.77 (0.49–0.90), *section V* = 0.76 (0.47–0.90), *section VI* = 0.89 (0.55–0.96).	Good (+)	SEM based on test-retest: *total* = 8.33, *section I* = 2.24, *section II* = 1.60, *section III* = 2.07, *section IV* = 4.46, *section V* = 0.69, *section VI* = 1.78; MDC_{95}: *total* = 22.82, *section I* = 6.06, *section II* = 4.38, *section III* = 5.67, *section IV* = 12.22, *section V* = 1.89, *section VI* = 4.87.	Good (-)

Rodrigues et al[39] (2014)	NA	NA	ICC (95% CI): *total* = 0.93 (0.80-0.98), *section I* = 0.94 (0.83-0.98), *section II* = 0.74 (0.25-0.91), *section III* = 0.88 (0.66-0.96), *section IV* = 0.72 (0.20-0.90), *section V* = 0.71 (0.16-0.90), *section VI* = 0.80 (0.43-0.93).	ICC (95% CI): *total* = 0.98 (0.94-0.99), *section I* = 0.95 (0.85-0.98), *section II* = 0.86 (0.64-0.96), *section III* = 0.90 (0.71-0.96), *section IV* = 0.85 (0.58-0.95), *section V* = 0.87 (0.68-0.96), *section VI* = 0.96 (0.89-0.99).	NA	Fair (+)	NA	NA
Sahin et al[40] (2019)	Cronbach's Alpha: *total* = 0.96.	Poor (+)	NA	NA	NA	NA	NA	NA

[a] AUC = Area under the Receiver Operating Characteristic curve; CFA = confirmatory factor analysis; ICC = intra-class correlation coefficients; MDC = minimal detectable difference; NA = not available; SEM = standard error of the mean.

Supplementary Figure 1. Meta-analysis forest plot displaying the estimate of test-retest (A) and inter-rater (B) reliability for BESTest total score from all intraclass correlation coefficients (ICC) reported in each study. The first column labels each article by the last name author's and year of publication. Error bars indicate 95% confidence intervals (CIs). Total = sample size.

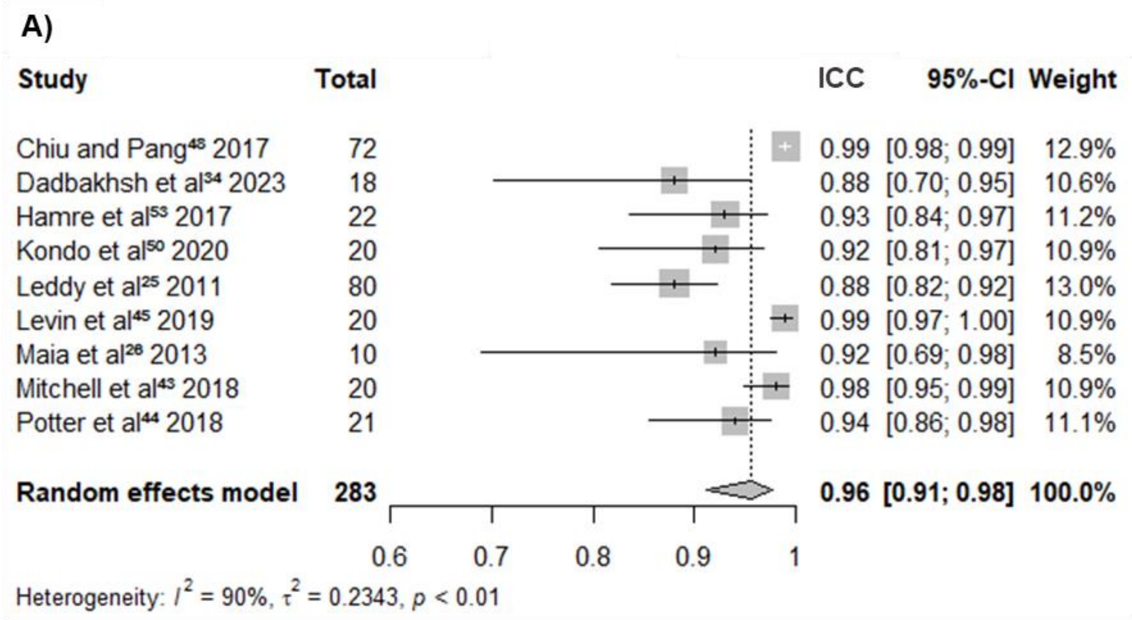

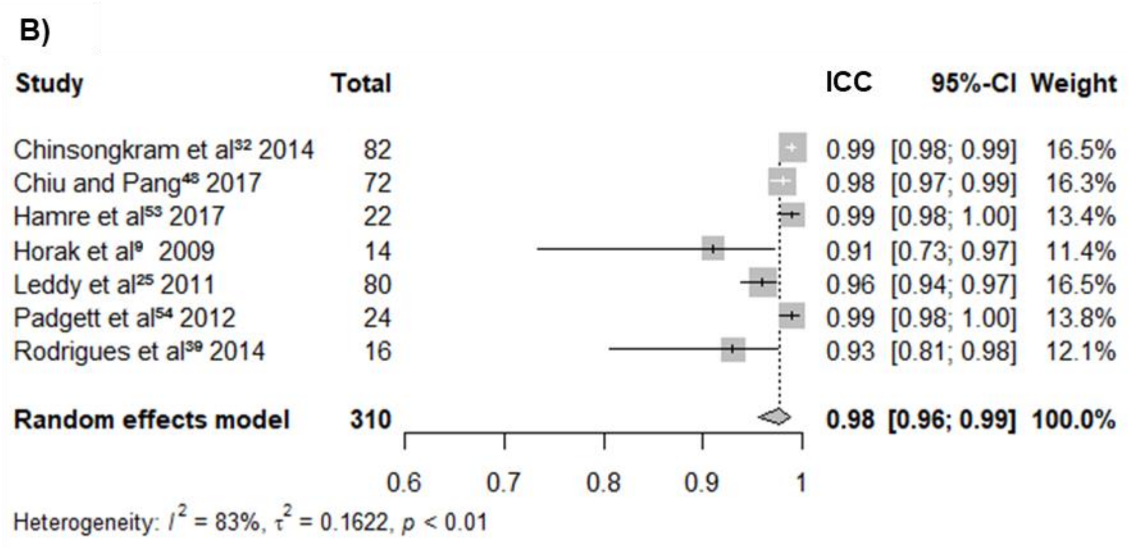

Supplementary Figure 2. Meta-analysis forest plot displaying the estimate of test-retest reliability for each section of the BESTest (A-F) from all intraclass correlation coefficients (ICC) reported in each study. The first column labels each article by the last name author's and year of publication. Error bars indicate 95% confidence intervals (CIs). Total = sample size.

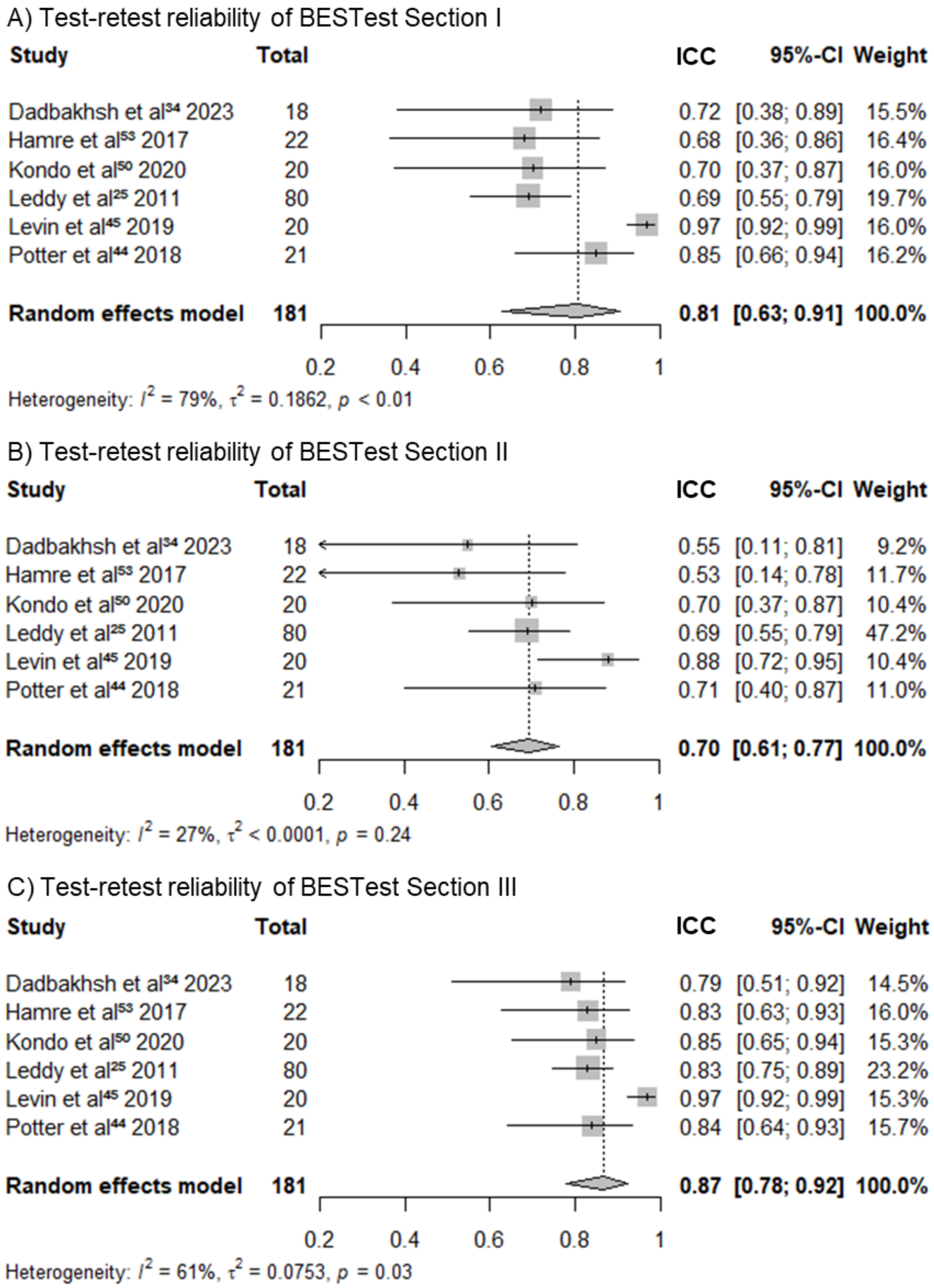

(continued)

Supplementary Figure 2. Continued

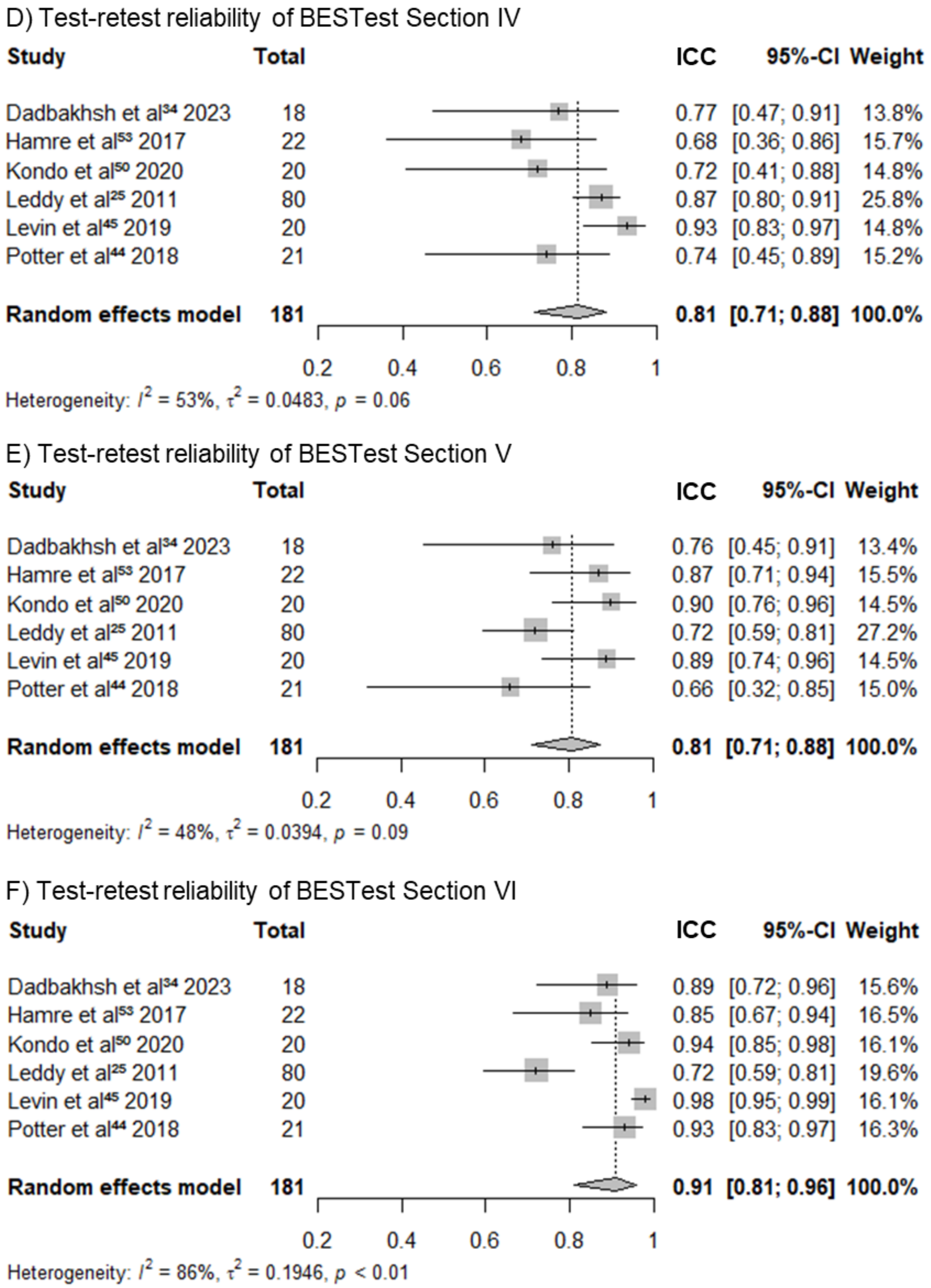

Supplementary Figure 3. Meta-analysis forest plot displaying the estimate of inter-rater reliability for each section of the BESTest (A-F) from all intraclass correlation coefficients (ICC) reported in each study. The first column labels each article by the last name author's and year of publication. Error bars indicate 95% confidence intervals (CIs). Total = sample size.

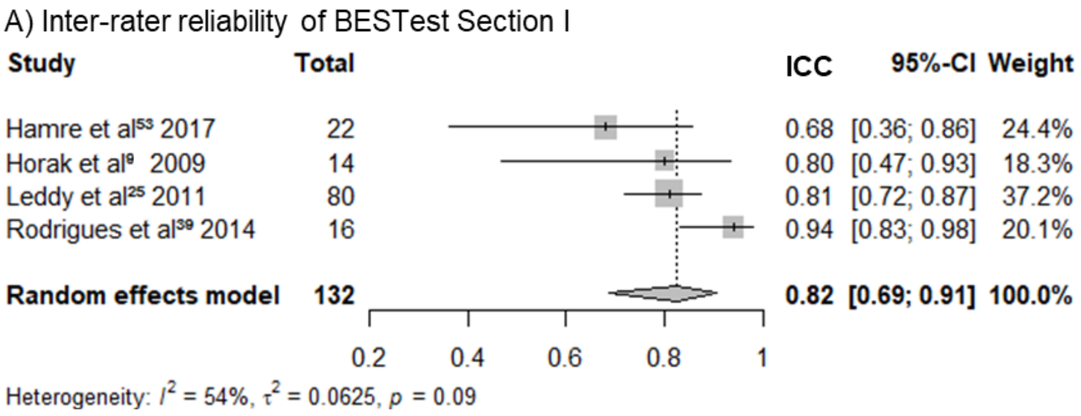

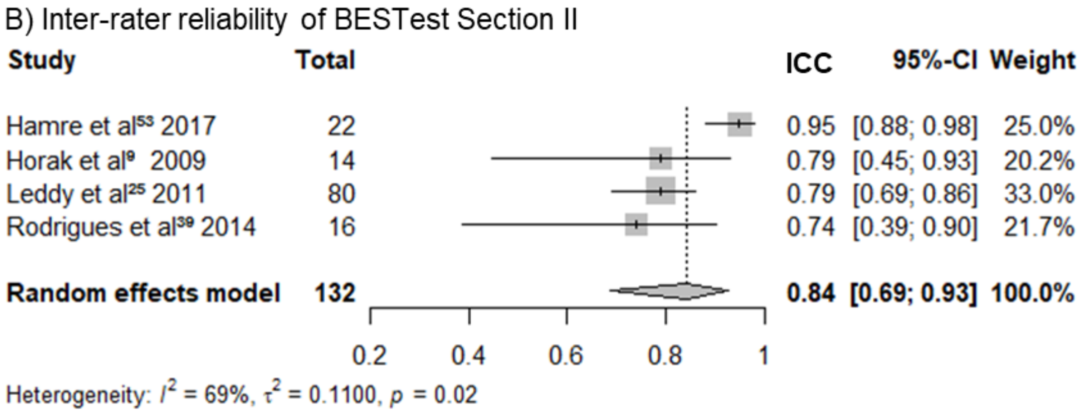

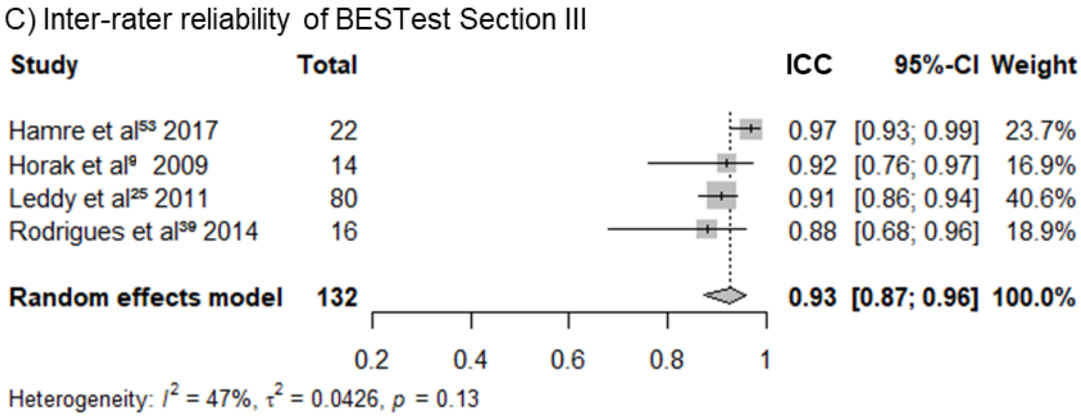

(continued)

Supplementary Figure 3. Continued

D) Inter-rater reliability of BESTest Section IV

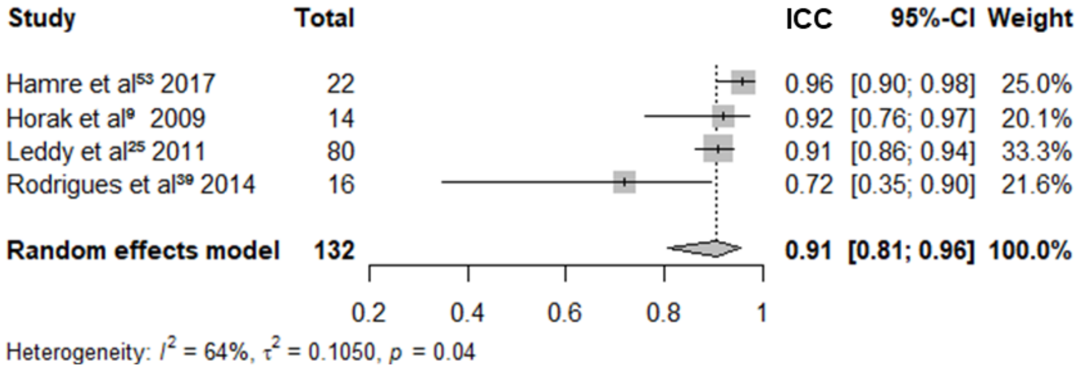

E) Inter-rater reliability of BESTest Section V

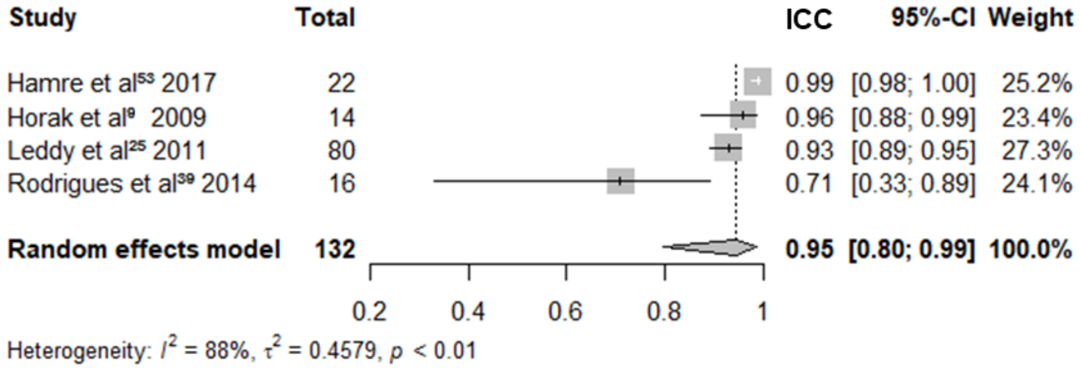

F) Inter-rater reliability of BESTest Section VI

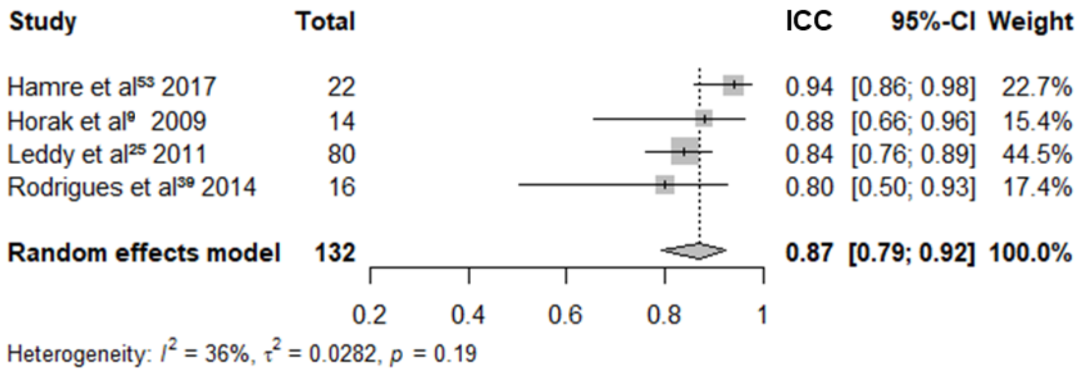

Supplement: 2023-0607_R2_Supplementary_Material_rev_pzae178 [file 2023-0607_r2_supplementary_material_rev_pzae178.pdf]
